# Image-Guided Magnetic Microrobots for Microplastic Capture and Biofilm Removal in the Female Reproductive System

**DOI:** 10.1021/acsnano.6c02432

**Published:** 2026-09-03

**Authors:** Fatma M. Yurtsever, Sagar Arya, Ester Maráková, Roshan Velluvakandy, Eva Odehnalová, Jakub Hejč, Zbyněk Heger, Martin Pumera

**Affiliations:** † Future Energy and Innovation Laboratory, Central European Institute of Technology, 48274 Brno University of Technology, Purkyňova 123, Brno 61200, Czech Republic; ‡ Department of Chemistry and Biochemistry, Mendel University in Brno, Zemědělská 1665/1, Brno 613 00, Czech Republic; § International Clinical Research Center, St. Anne’s University Hospital Brno, Pekařská 664/53, Brno 602 00, Czech Republic; ∥ Center of Advanced Innovation Technologies, Faculty of Materials Science and Technology, VSB - Technical University of Ostrava, Ostrava CZ-708 00, Czech Republic; ⊥ Department of Medical Research, China Medical University Hospital, China Medical University, No. 91 Hsueh-Shih Road, Taichung 40402, Taiwan; # Office of Research and Development, Asia University, Taichung 40402, Taiwan; ∇ Faculty of Electrical Engineering and Computer Science, VSB - Technical University of Ostrava, 17. Listopadu 2172/15, Ostrava 70800, Czech Republic

**Keywords:** microrobots, female reproductive system, biofilm removal, microplastic capture

## Abstract

The health of the female reproductive system is increasingly threatened by emerging environmental contaminants that cause potential bacterial infections, cancer, and fertility problems. Yet effective strategies for the localized removal of these contaminants remain largely unexplored. Among these contaminants, microplastics have emerged as a major global health concern, with growing evidence of their accumulation in human tissues and associated with infectious diseases, carcinogenesis, cellular aging, and infertility due to direct or indirect contact with synthetic fabrics, plastic kitchenware, and disposable feminine hygiene products. Despite these risks, the removal of retained microplastics, particularly from sensitive reproductive environments, remains a significant challenge in biomedical research. Here, we report an image-guided magnetic soft microrobot platform designed for targeted microplastic capture and biofilm eradication within the female reproductive system. Magnetic soft microrobots were fabricated using a droplet solidification method and functionalized with a polydopamine coating via self-polymerization to enhance the surface adhesion and capture efficiency. Real-time visualization and precise navigation were enabled under X-ray fluoroscopy through the incorporation of tantalum-based contrast agents. Functionally, the magnetic soft microrobots inhibited up to 67% of biofilm growth and achieved microplastic capture efficiencies of up to 80% in phosphate-buffered saline and 50% in simulated vaginal fluid, demonstrating a robust performance under biologically relevant conditions. This study demonstrates the integration and application of image-guided microrobots for the removal of microplastics and biofilms in the female reproductive system, offering a promising strategy for minimally invasive interventions to protect reproductive health from environmental pollutants.

## Graphical abstract

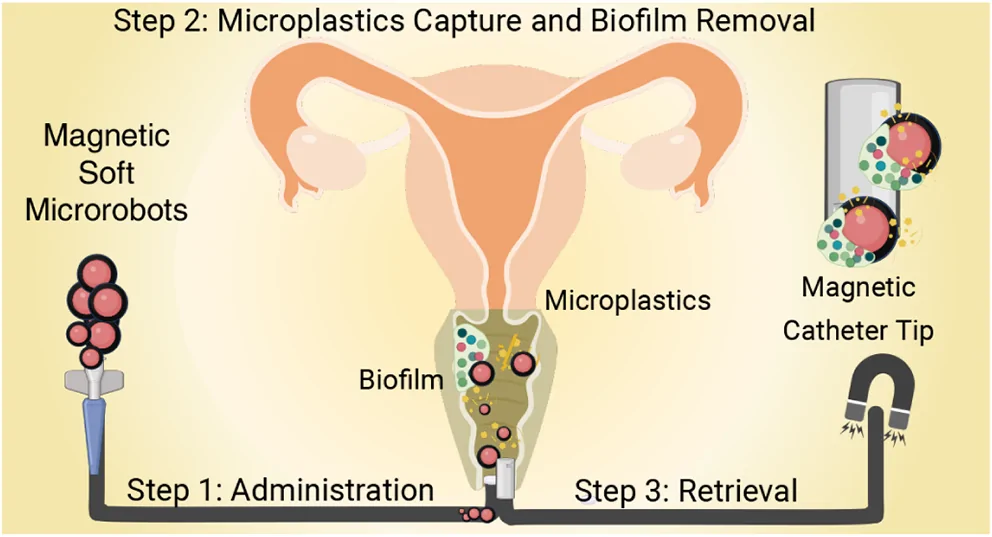

## 1 Introduction

Microplastics and nanoplastics are one of the major concerns that threaten public health and environmental safety. Global plastic manufacturing and consumption have increased dramatically over the past century, reaching over 350 million tons and expecting 1.2 billion tons by 2060.^1–4^ Recent studies have revealed that microplastics and nanoplastics have been found in several organs and body fluids, including the lungs, brain, uterus, placenta, blood, breast milk, and saliva, as illustrated in Scheme 1A.^5–11^ Microplastics (typically 1 μm to 5 mm) and nanoplastics (<1 μm) can enter the human body primarily through inhalation and ingestion, with dermal contact representing a potential but more limited route, particularly for particles smaller than approximately 10 μm (and especially nanoplastics).^12,13^ It has been reported that microplastics and nanoplastics accumulate inside the organs, causing cytotoxicity, oxidative stress, immunogenicity, endocrine, cardiovascular, and pulmonary disruption, and cardiovascular as well as infectious diseases.^1,14^ They raise significant concern regarding reproductive toxicity. Emerging evidence from experimental models indicates adverse effects on ovarian function, folliculogenesis, hormone levels, and fertility, with potential biological, intergenerational, and broader societal implications for reproductive health.^15,16^ Animal studies have shown that microplastics cause pathological alterations in the uterus, causing uterine fibrosis, inflammation, a decrease of uterine glands and endometrial thinning, reduced oocyte maturation, and fertility issues.^17–20^ Reports indicate that 12 of 24 feminine hygiene products (in particular tampons) contain plastics that are directly in contact with vaginal walls, which release 17 billion nanoplastic fibers during *ex vivo* tests.^21^ Microplastics and nanoplastics have the potential to host pathogens, including bacteria, fungi, and viruses, causing infectious diseases, particularly in the female reproductive system, as shown in Scheme 1B.^22,23^ It has been reported that microplastic-associated biofilm formation induces antimicrobial resistance genes, which is one of the major issues in public healthcare settings.^24,25^ Therefore, the targeted, remote, and noninvasive removal of microplastics from the female reproductive system is highly critical to minimize biofilm formation and bacterial infections and to prevent other diseases.

**Scheme 1:**
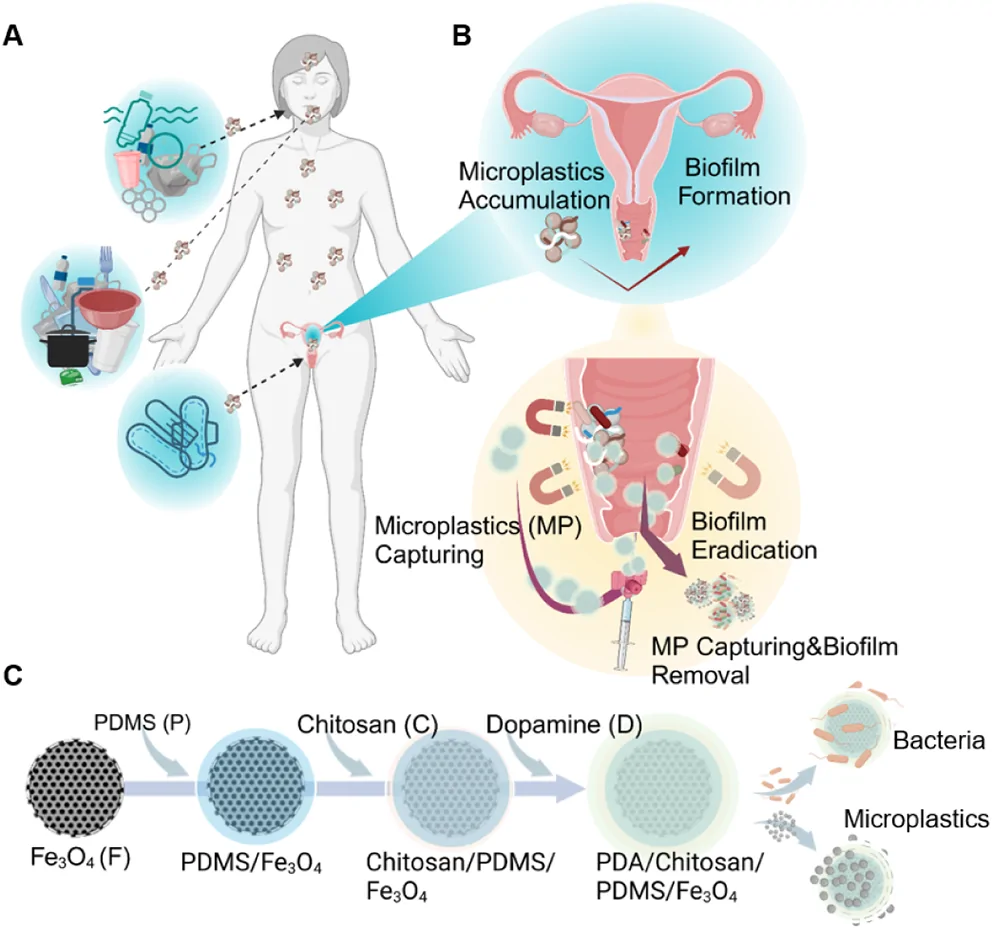
Illustration of Microplastic Exposure, Accumulation, in Female Reproductive System and Therapeutic Intervention Using Presented Microrobots^a^

a A) Sources of microplastics are found in the human brain, kidney, liver, gastrointestinal system, as well as the female reproductive system. Scheme also illustrates the capturing of microplastics and the eradication of biofilm using magnetic soft microrobots. Scheme and illustration were prepared with BioRender.com. B) Microplastics accumulate in the female reproductive tract, inducing bacterial colonization and biofilm formation. The proposed therapeutic approach uses polydopamine-coated magnetic soft microrobots to capture microplastics and eradicate biofilms. Design of the magnetic soft microrobots. C) Stepwise synthesis of the magnetic soft microrobots used for biofilm removal and microplastic capture.

In this study, we demonstrated the ability to capture microplastics and eradicate biofilms in a simulated biological environment of the female reproductive system by integrating different functionalities in image-guided magnetically actuated soft microrobots. The method employs a cost-effective and straightforward droplet solidification approach by using a homogenizer. These soft microrobots were additionally incorporated with a contrast agent for visualization under medical, guided, and real-time imaging using X-ray fluoroscopy. Previously, polydopamine-coated magnetic nanorobots (MagRobots) decorated with lipase enzymes were tested for capturing and degrading polycaprolactone (PCL)-based microplastics.^26^ It was reported that the adhesive properties of MagRobots helped capture PCL microplastics, which were enzymatically degraded after 24 h of incubation.^26^ In this study, polystyrene (PS) microplastics were selected as a model particle due to their widespread environmental prevalence and common human exposure through food, water, and air. Previous research in the literature has demonstrated their ability to accumulate in uterine and endometrial tissues.^8^ Here, we employed magnetic soft microrobots to demonstrate *ex vivo* microplastic capture, quantification using fluorescence-labeled, carboxylate-modified polystyrene microplastics, biofilm removal, actuation, imaging in a phantom model using a rat uterine horn, and retrievability in a 3D-printed vasculature model.

## 2 Results and Discussion

### 2.1 Soft Microrobot Design, Fabrication, and Characterization

Magnetically actuated soft microrobots were developed to facilitate microplastic capture and biofilm eradication. The microrobots were surface-functionalized by polydopamine coating via the previously reported self-polymerization method for ease of functionalization and adhesion.^27–29^ Enhancement of the ionic interaction was accomplished by incorporating positively charged chitosan into the soft scaffold. It was incorporated with commercially available 2–3 μm-sized tantalum (Ta) nanoparticles for medical imaging with X-ray fluoroscopy. The concentration of the tantalum was changed to evaluate the effect of tantalum in the actuation of the microrobots, which is shown in the following section. These soft microrobots were successfully prepared using a droplet solidification process within the oil–water emulsion by applying stronger shear forces to the polydimethylsiloxane (PDMS)-based soft scaffold in a 2% poly­(vinyl alcohol) (PVA) solution using a homogenizer as shown in Figure S1. Different scales of spherically shaped microrobots were formed, as shown in Figure 1A. Scanning electron microscopy (SEM) images in Figure 1B show the morphology of the uncoated (left) and coated (right) soft microrobots.

**Figure 1:**
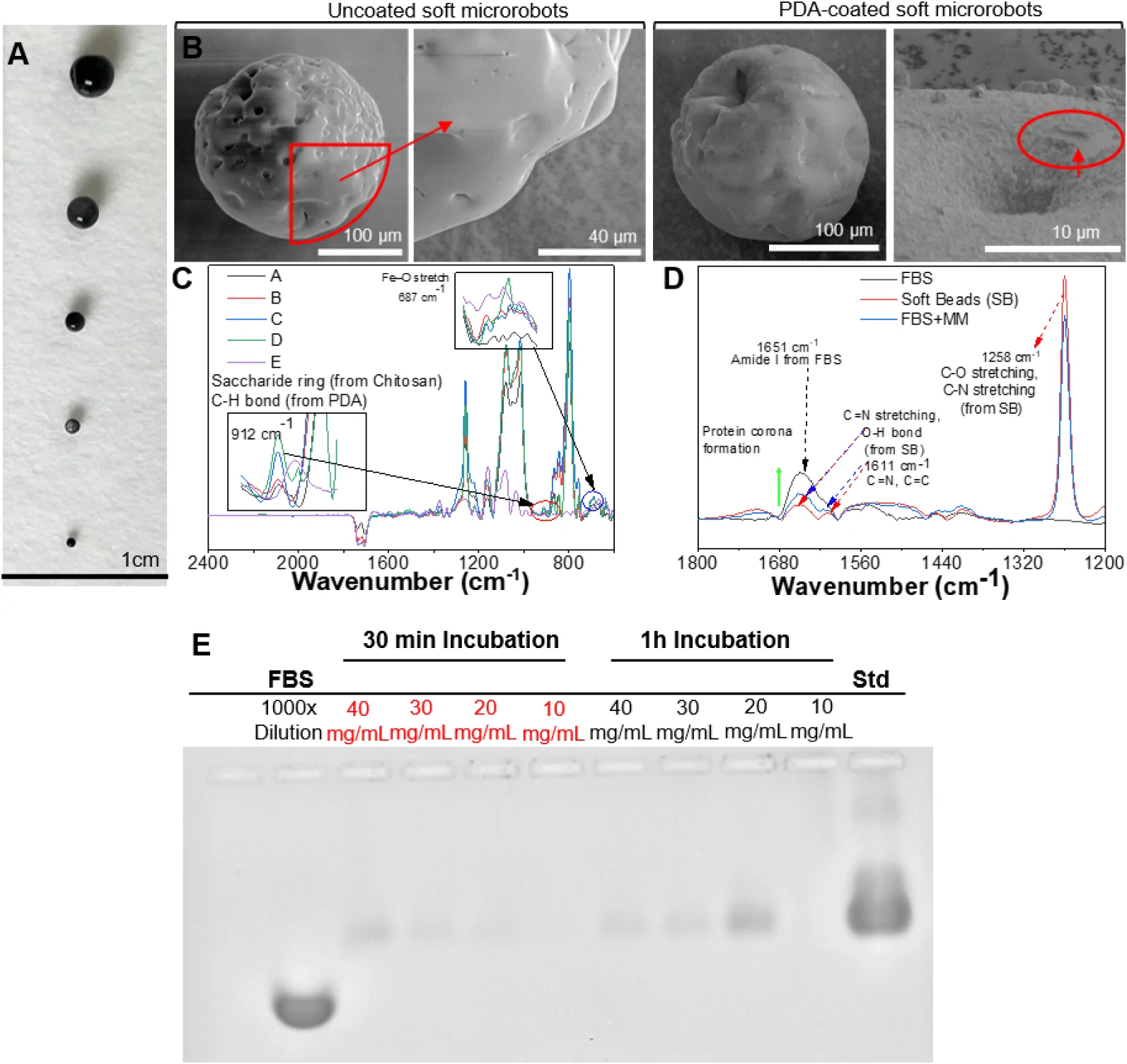
Soft microrobots’ morphology and characterization. A) Photograph of different-scale soft microrobots captured using a smartphone. Morphological analysis. B) SEM images of uncoated (left) and PDA-coated soft microrobots (right). C) FTIR spectra of soft microrobot components: PDMS (A), PDMS and Fe_3_O_4_ (B), PDMS, Fe_3_O_4_, and chitosan (C), PDMS, Fe_3_O_4_, chitosan, and polydopamine (D), and chitosan only (E). Investigation of protein corona formation. D) FTIR spectra of FBS, soft microrobots, and protein corona formation on soft microrobots. E) Gel electrophoresis showing protein corona formation on various doses of magnetic soft microrobots after 30 min and 1 h of incubation in FBS.

Fourier-transform infrared spectroscopy (FTIR) measurements were conducted to evaluate possible interaction between soft scaffold components including polydimethylsiloxane (PDMS) (A), PDMS + Fe_3_O_4_ (B), PDMS, Fe_3_O_4_, and chitosan (C), PDMS, Fe_3_O_4_, chitosan, and polydopamine (D), and chitosan only (E). FTIR spectra in Figure 1C show the PDMS+ Fe_3_O_4_ scaffold at 1260, 1040, 800–860, and 687 cm^–1^, corresponding to CH_3_, O–Si–O, Si–C, and Fe–O, respectively. The peak spectra at 912 cm^–1^ indicate the presence of C–H bonding from PDA and the saccharide ring from chitosan in the corresponding C and D lines. Next, we evaluated the protein corona formation using the FTIR technique. Figure 1D represents the FTIR spectra of the soft microrobots in fetal bovine serum (FBS). In the spectrum, the blue line presents a corresponding amide I peak in FBS at 1651 cm^–1^ while the red line corresponding to the soft microrobots with a PDA coating layer indicates C=N stretching and O–H bonding at 1651 cm^–1^. The increased peak intensity indicated the presence of a protein corona on the soft microrobots’ surface. This was further corroborated by gel electrophoresis, which confirmed the formation of protein coronas after both 30 min and 1 h incubations in all tested concentrations of microrobots except for the lowest tested dose (10 mg/mL) (Figure 1E).

### 2.2 Magnetic Motion of the Soft Microrobots inside the Phantom Model

Incorporating magnetite in the soft microrobots enabled their successful activation using an external magnetic field generator. Actuation of the soft microrobots was evaluated in simulated vaginal fluid (SVF) with or without the addition of 1.5% mucin to mimic the viscous environment of vaginal fluid. The trajectories of the microrobots are shown in Figure 2A (top and bottom). Prior to the actuation studies, magnetic properties were evaluated using a vibrating sample magnetometer (VSM). A magnetic hysteresis loop of soft magnetic microrobots indicates superparamagnetic behavior as shown in Figure S2. The mean speed of the soft microrobots was recorded for various concentrations of the contrast agent as shown in Figure 2B. High standard deviation might be relevant to the size difference of the microrobots, which results in different amounts of magnetite loading. It has been recorded that the viscosity by adding the mucin to the SVF (actuation medium) reduces the actuation of the microrobots by around 10-fold. Next, the soft microrobots were delivered inside the uterine horns of a female rat using a butterfly syringe needle. The actuation was controlled by a permanent neodymium–iron–boron (NdFeB) magnet as shown in Supplementary Video S1. Time loops of the motion at 0, 2.5, 5, and 10 s are shown in Figure 2C (i–iv), respectively.

**Figure 2:**
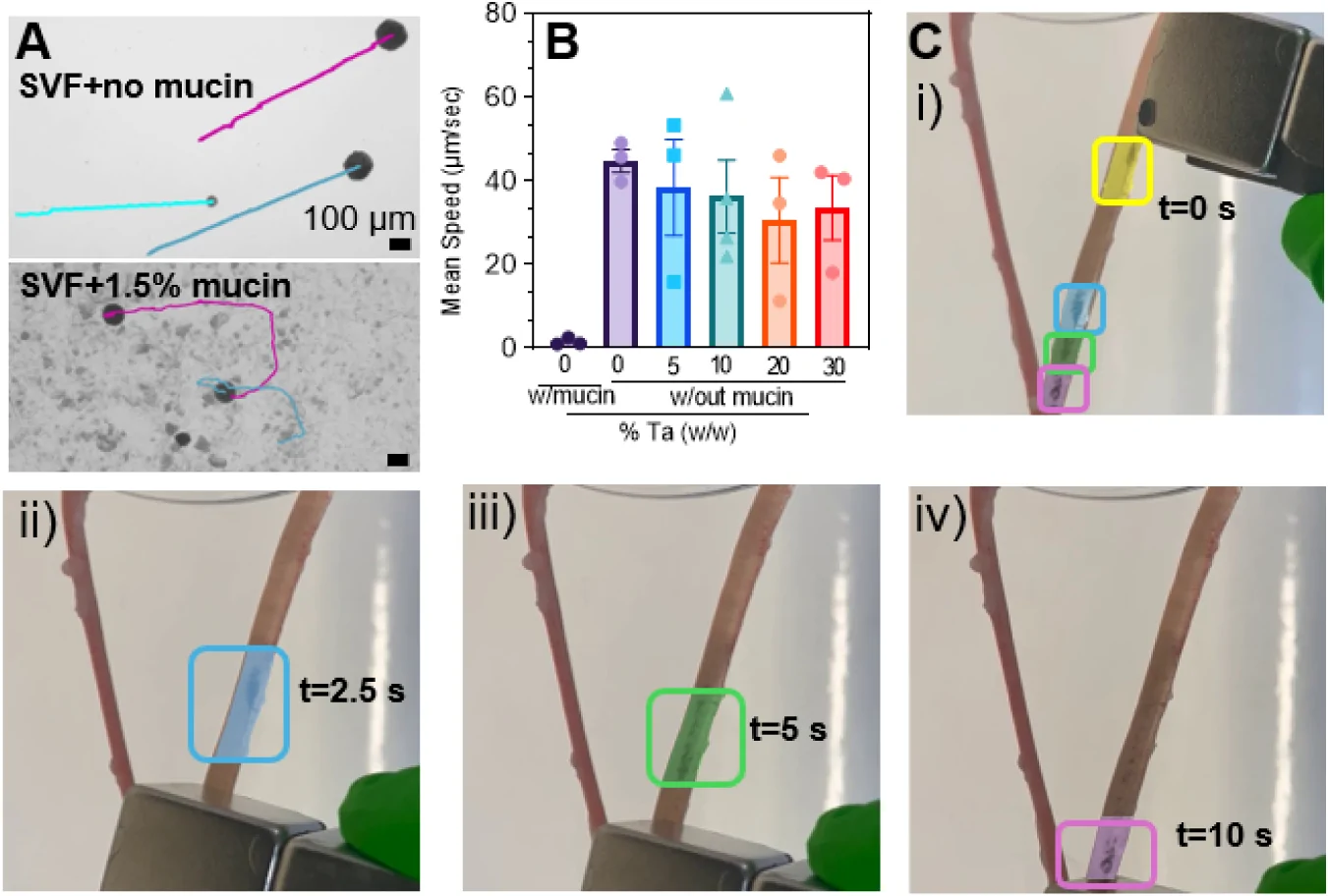
Motion analysis. A) Trajectory of the magnetic soft microrobots in simulated vaginal fluid with no mucin (top) and with 1.5% mucin (bottom). B) Histogram of the motion analysis of the magnetic soft microrobots, including different amounts of contrast agent, in the presence and absence of mucin under a magnetic field of 5 mT with the frequency of 10 mHz in SVF. Data shown are the averages of three replicates. C) Time-lapse images of externally controlled motion of the soft microrobots in the female rat uterine horn. Images are shown in (i) 0 s, (ii) 2.5 s, (iii) 5 s, and (iv) 10 s.

### 2.3 Quantification of Microplastic Capture, Biofilm Eradication, and Medical Imaging

To quantify the microplastic capture, we first examined the interaction between the soft microrobot and fluorescence-labeled carboxylate-modified polystyrene microplastics. The microplastic-capturing experiment setup is illustrated in Figure 3A. The optical microscope image in Figure 3B shows that the soft microrobots successfully captured the micron-sized microplastics on their surface. To validate capture, the trajectory was recorded in different fluids over time as shown in Supplementary Videos S2 and S3. Furthermore, the SEM images in Figure 3C confirm the presence of microplastics captured on the surface of the soft microrobots. Next, quantitative studies were conducted by using a spectrofluorometer. The emission spectra in phosphate-buffered saline (PBS) and SVF medium are shown in Figure 3D and 3F. Fluorescence intensity decreased over time, indicating that microplastics were captured and removed in both media. Quantitative analysis of the remaining microplastics is shown in the histograms in Figure 3E and 3G. Total microplastic capture reached up to 80% in PBS medium compared to around 50% in SVF. The reduced capture rate in the SVF may be attributed to its higher ionic strength. The capturing capacity per microrobot was calculated using the captured microplastic particles per microrobot. In 30 min, 7.82 × 10^4^ particles/microrobot were obtained, and in 1 h, it reached up to 1.07 × 10^5^ particles/microrobot, while in 2 and 4 h, the total capture capacity was reached up to 1.44 × 10^5^ and 1.58 × 10^5^ particles/microrobot in PBS. The capacity is limited by the total available surface area of the microrobots. Afterward, microplastic capturing and releasing were observed before and after the washing step with DI water as shown in Figure 3H. Furthermore, qualitative studies have demonstrated the capture of the cotton microfibers released from feminine hygiene pads in addition to quantitative studies on the capture of polystyrene-based microplastics. The cotton microfibers were adsorbed and carried by the microrobots depending on the length of the fibers as shown in Figure 3I, demonstrating that the capture mechanism is not restricted by the spherical particles. However, the capture of cotton-based microfibers was not as high as that of polystyrene-based microplastics. The corresponding videos are recorded at Supplementary Video S4.

**Figure 3:**
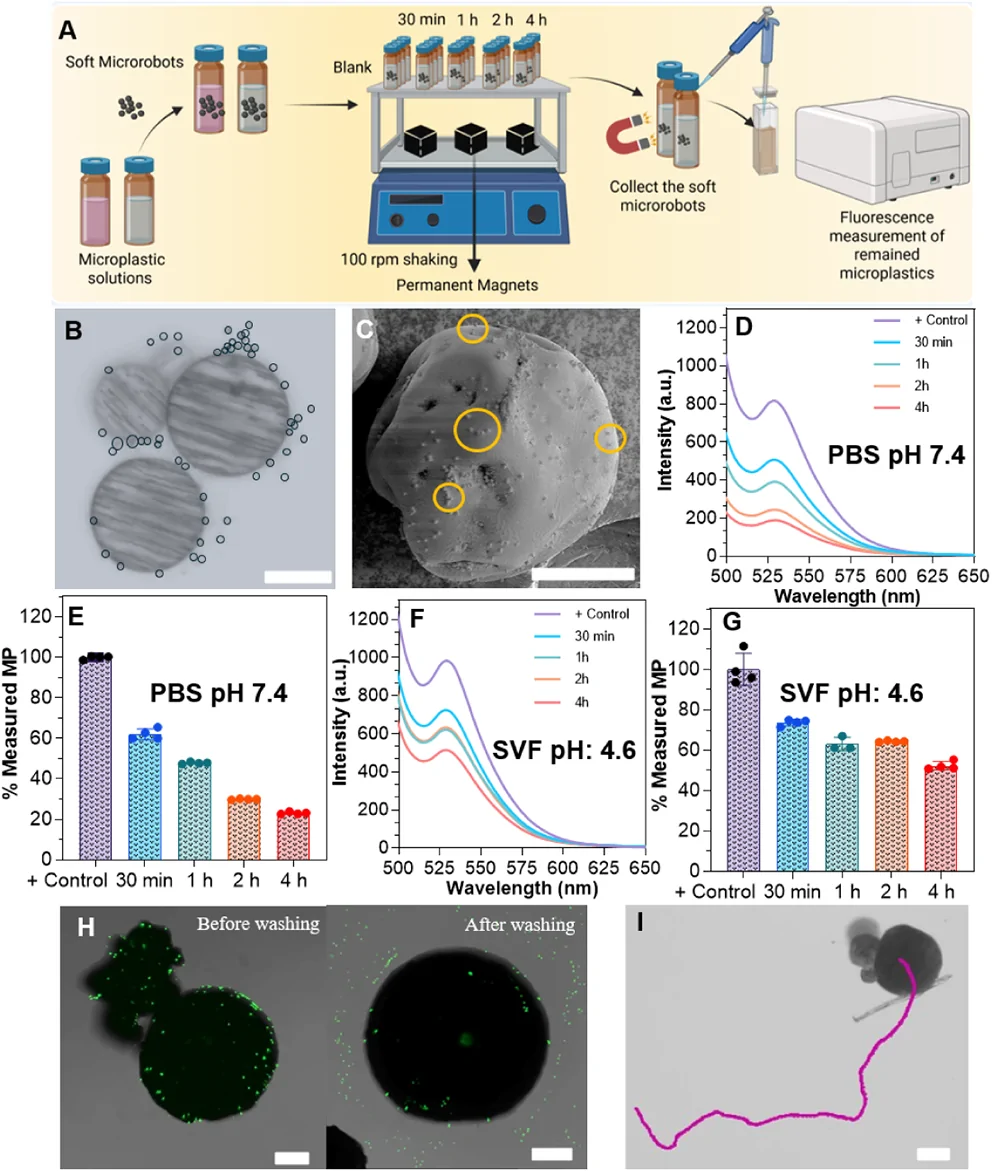
Qualitative and quantitative microplastic-capturing studies. A) Schematic illustration of microplastic capturing using soft microrobots and quantification studies using a spectrofluorometer. Schemes and illustrations were prepared with BioRender.com. Morphological analysis. B) Optical microscopy image of microplastic capture by a magnetic soft microrobot in SVF (scale bar: 50 μm). C) SEM image of captured microplastics on magnetic soft microrobots. Quantification studies. D) Emission spectra of fluorescence-active microplastics (carboxylate-modified polystyrene) in pH 7.4 PBS. E) Histogram of time-dependent studies of microplastic remaining in PBS at pH of 7.4. F) Emission spectra of fluorescence-active microplastics (carboxylate-modified polystyrene) in SVF at pH 4.6. G) Histogram of time-dependent studies of microplastics remaining in SVF at pH 4.6. H) Fluorescence microscopy imaging of magnetic soft microrobots before and after washing to evaluate capture and removal on the surface of the microrobots. I) Trajectory of the magnetic microrobots carrying microfiber released from accelerated agitation of a menstrual hygiene pad.

After demonstrating microplastic capture in two different simulated mediums, we next investigated the soft microrobots’ interactions with bacterial suspensions and biofilm eradication as illustrated in Figure 4A and 4C. The percentage of the remaining biofilm was calculated with the equation:

$$\%\mathrm{Remained Biofilm or}\;\mathrm{E. coli}=\frac{Ac-At}{Ac}\times 100$$

where *Ac* is the absorbance of the bacteria only as control group without any treatment after background subtraction, and *At* is the absorbance at the treatment time. Figure 4B shows that a 25% capture efficiency was achieved after 4 h of treatment of the planktonic bacteria. Representative SEM images of the captured bacteria on the surface of the soft microrobot are shown in Figure S4. The evaluation of biofilm eradication was further assessed by magnetically actuated microrobots on a mature biofilm, followed by measuring the optical density of the remaining biofilm over a period of 4 h using Crystal Violet staining. Representative inset images indicate heatmap profiles of remaining biofilm, and histograms are shown in Figure 4D. Biofilm eradication reached 67% after a 4 h treatment time. Subsequently, bacterial viability was evaluated using live/dead fluorescence staining to assess the antibacterial activity of the microrobots. Bright-field (BF) and fluorescence micrographs are shown in Figure 4E. The green fluorescence represents live bacteria adsorbed onto the microrobot surface after a gentle wash, while the red stain indicates the dead bacteria on the microrobot surface. The interference of the microrobots and the staining was tested before the time-dependent bacterial-viability test. No interference was observed between the microrobots and the SYTO 9/PI dyes, as the control experiments with microrobots alone confirmed that the live/dead stain interacts specifically with the bacterial strain and does not produce fluorescence signals from the microrobots (Microrobot Only control). No bacterial capture was observed on the microrobots without PDA coating. These results indicate that the PDA coating promotes bacterial attachment and increased bacterial death, and the number of dead bacteria increased with treatment time. It is also shown that as the treatment time increases, the bacterial adsorption and the amount of dead bacteria increase.

**Figure 4:**
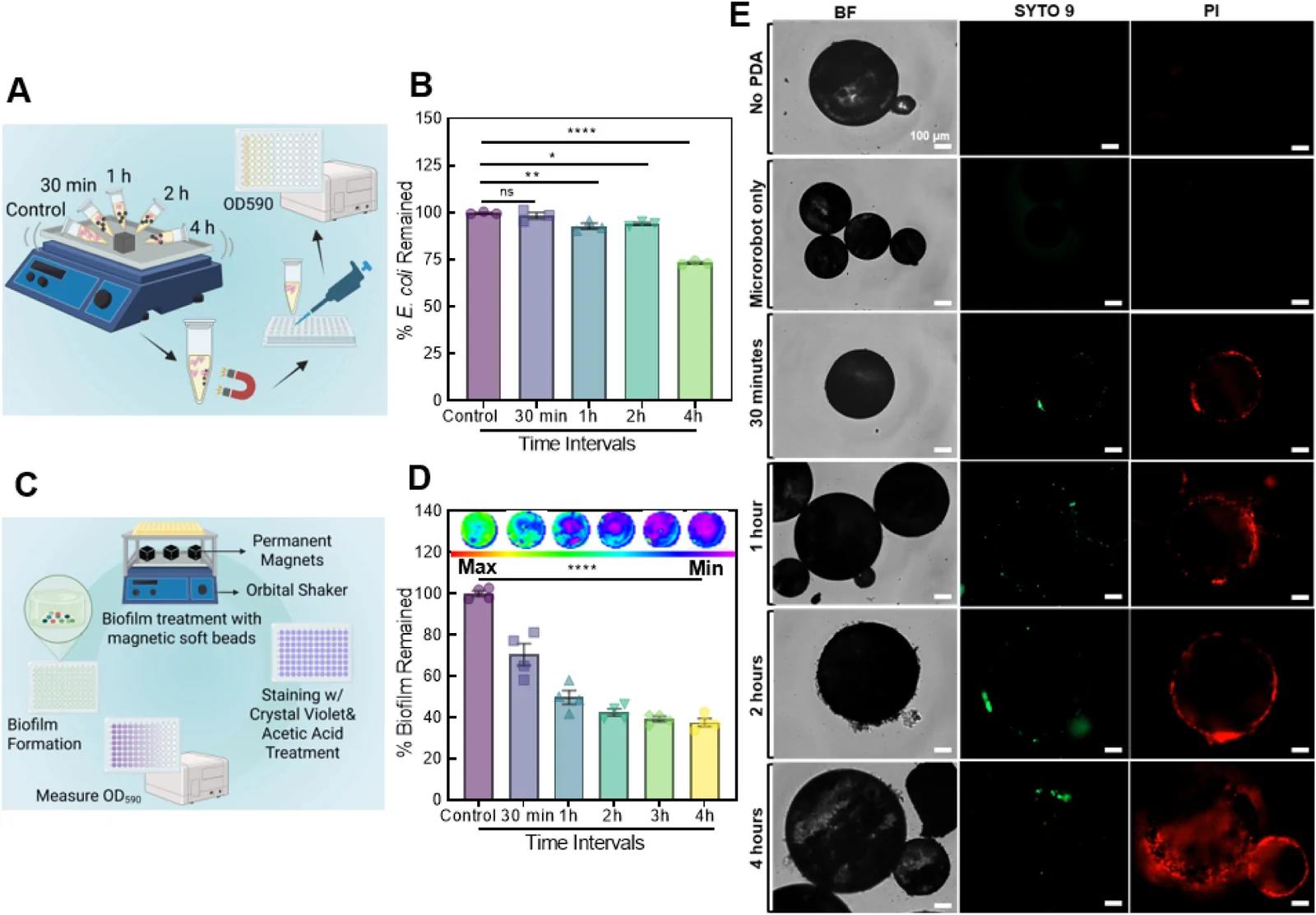
*Ex vivo* biofilm eradication studies and medical imaging. A) Illustration of bacterial-capture experiment. Illustration was prepared with BioRender.com. B) Histogram of the percentage of remaining *E. coli* on time-dependent turbidity measurements at 590 nm. C) Illustration of the biofilm eradication setup. Illustration was prepared with BioRender.com. D) Percentage of remaining biofilm after Crystal Violet staining. Inset images represent the heatmap of remaining biofilm after eradication on a 96-well plate at different time intervals. E) Fluorescence images of captured *E. coli* on the surface of the microrobots under different treatment times, including 30 min, 1, 2, and 4 h. Two different control groups were demonstrated without PDA coating and without bacteria to obtain the effect of PDA surface on bacterial killing and any interference between staining and microrobots. Data shown are the averages of three replicates (panel B) and four replicates (panel D). Full dataset and heatmap profiles used in panel D can be found in Figure S5 in the Supporting Information. The standard error of the mean is represented by error bars. A one-way ANOVA test was conducted for statistical analysis. *P < 0.05, **P < 0.01, ***P < 0.001, and ****P < 0.0001 indicate statistical significance compared with the untreated bacterial suspension as a positive control and soft microrobots without a contrast agent.

To assess the potential of the soft microrobots for clinically relevant imaging, we demonstrated real-time visualization in both a syringe and a “phantom” rat uterine horn using X-ray fluoroscopy. The experimental setup is illustrated in Figure S6. The intensity of the fluoroscopy images of syringes is shown in Figure 5A. The soft microrobots without Ta as a contrast agent in the syringe were not visible under X-ray fluoroscopy (second column), whereas the incorporation of more than 20% (w/v) Ta enables clear visualization of the soft microrobots. In order to maintain the significant visualization, the contrast agent concentration was fixed to 30% (w/v). Next, the soft microrobots and saline were injected into the *ex vivo* rat uterine horns using a butterfly syringe needle (Figure 5B). As shown in Figure 5C, the incorporation of a contrast agent enabled visualization of the soft microrobots using X-ray fluoroscopy within the phantom model. The retrieval of the microrobots was demonstrated using a magnetic-tip endovascular transcatheter in a phantom vasculature model in Supplementary Video S4. In order to support the retrieval, a time-lapse of the retrieval was shown in Figure 5D and Supplementary Videos S5 and S6.

**Figure 5:**
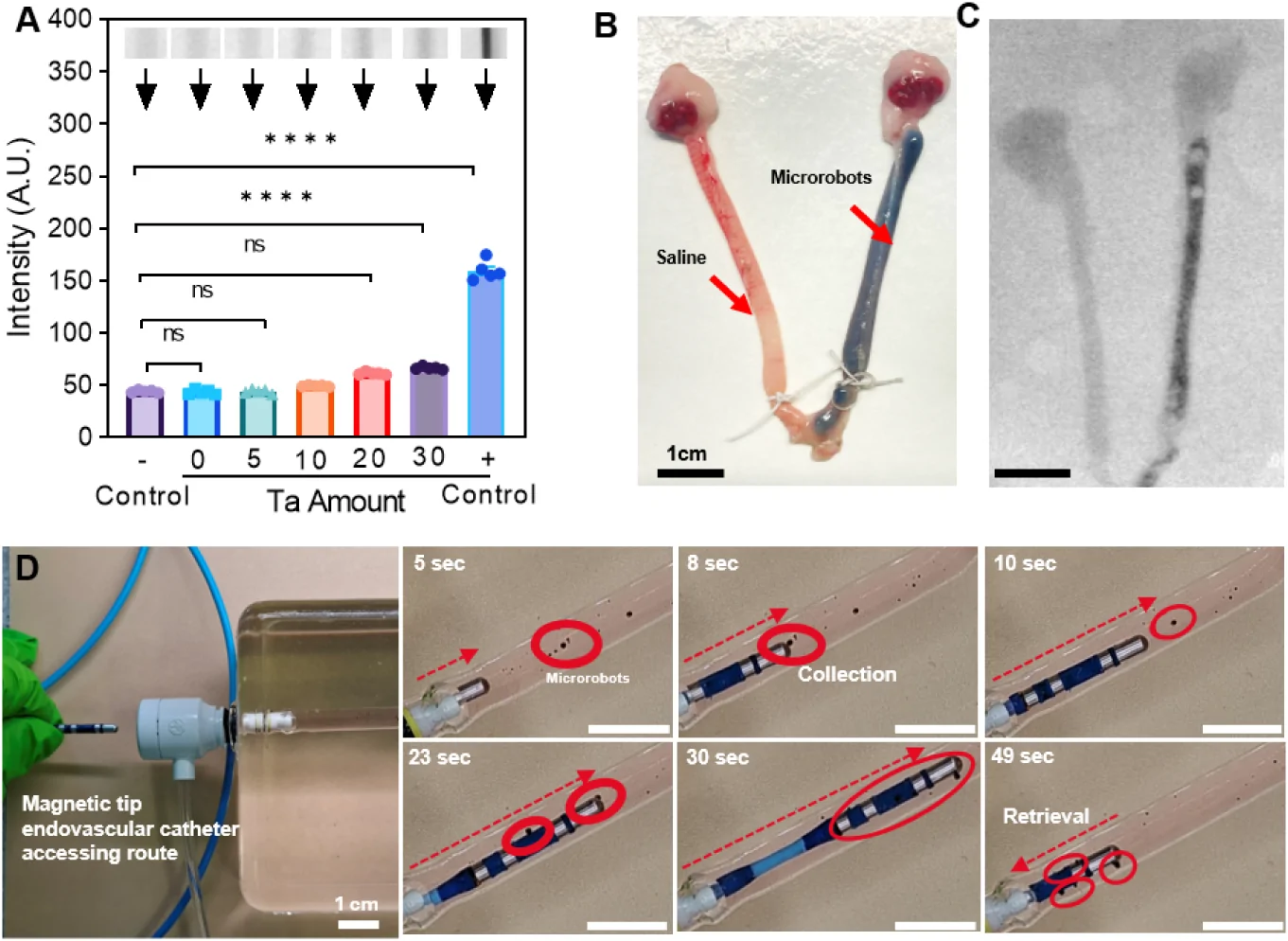
Real-time medical imaging. A) Histogram of X-ray fluoroscopy intensities as recorded for (from left to right): X-ray fluoroscopy images of syringes including saline as negative control, soft microrobots without contrast agent, soft microrobots with various concentrations of Ta, the contrast agent, and only contrast agent containing 30% (w/v) Ta. B) Photograph of saline-injected rat uterine horns (left) and soft microrobots (right). C) Real-time medical imaging of the soft microrobots under X-ray fluoroscopy connected with the C-arm setup, comparing saline-injected (left) and magnetic soft microrobots (right) in the rat uterine horns. D) Demonstration of the retrievability of the magnetic soft microrobots using a magnetic-tipped endovascular catheter. Data shown are the averages of five replicates (panel A). The standard error of the mean is represented by error bars. A one-way ANOVA test was conducted for statistical analysis. *P < 0.05, **P < 0.01, ***P < 0.001, and ****P < 0.0001 indicate statistical significance compared to the 30% w/v Ta contrast agent in saline.

To better understand the potential cytotoxicity of magnetic soft microrobots, an MTS assay was performed using the human fibrosarcoma epithelial-like cell line (HT1080). Results in Figure 6A indicate that the magnetic soft microrobots are nontoxic after incubation for 24 and 48 h. To confirm this finding, viability was assessed by measuring the ATP level in the HEK-293 cells. The results in Figure 6B showed similar trends to the MTS assay with four different concentrations that are nontoxic. Magnetite nanoparticles (Fe_3_O_4_) only were used as control groups with similar concentrations showing relatively high cytotoxicity in HEK-293 cells (Figure S7). This indicates the minimal leaching of magnetite nanoparticles as the microrobots remained nontoxic to the cells throughout the whole testing period. To investigate the effect of soft microrobots on proliferation, HEK-293 cells were exposed to the various doses of magnetic soft microrobots for 24 and 48 h, and then the cells were stained with bromodeoxyuridine (BrdU). The proliferation was not significantly affected by magnetic soft microrobots (Figure 6C). The high standard deviation might be explained by the attachment of the microrobots to the cells and washing them away during multiple washing steps. Afterward, reactive oxygen species (ROS) production was evaluated, as ROS act as crucial molecules triggering and amplifying inflammation processes. We found that there is no ROS generation throughout the tested doses of microrobot exposure (Figure 6D). Although the results did not directly assess tissue inflammation, mucosal integrity, or the inflammatory response, they provide critical evidence that microrobots do not induce cytotoxicity, suppress proliferation, or induce oxidative stress. Taken together, the obtained results clearly demonstrate that soft microrobots exhibit an exceptional biocompatibility.

**Figure 6:**
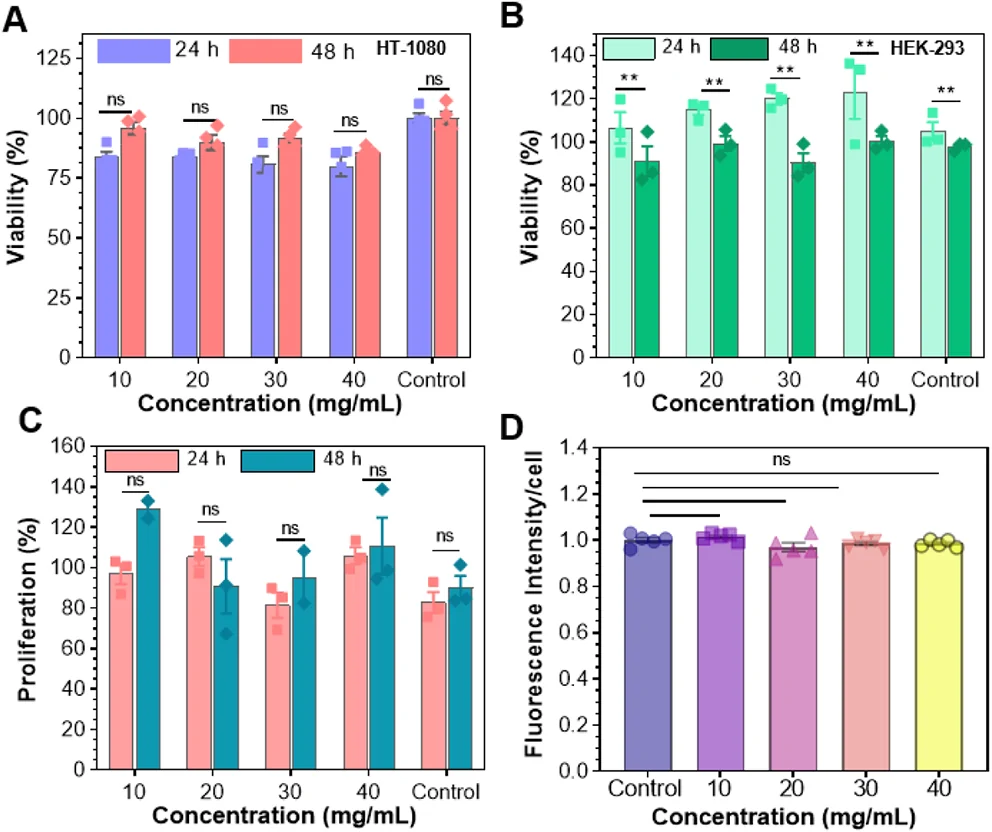
Biocompatibility of the magnetic soft microrobots. A) Viability of human HT-1080 and B) HEK-293 cell lines after incubation with varying doses of magnetic soft microrobots for 24 and 48 h. C) Proliferation analysis indicating that HEK-293 cells were not significantly affected by various doses of magnetic soft microrobots. D) Intracellular ROS quantification in HEK-293 cells. The standard error of the mean is represented by error bars. The data shown are the averages of three replicates in panels A, B, and C and 4 replicates in panel D. *P < 0.05 versus treatment only unpaired *t*-test.

### 2.4 Study Limitations

Despite demonstrating delivery, actuation, clinically relevant imaging, retrievability, biocompatibility, and promising application for microplastic capture and biofilm eradication in the female reproductive system using image-guided magnetic soft microrobots, the study is limited to making assessments of simulated and *ex vivo* models. The efficiency of microplastic capture and biofilm removal under *in vivo* conditions may result in a low yield. Before clinical applications, it is highly critical to expand the work to broader implications by evaluating different bacterial strains in complex microbial environments and microplastics with different morphologies, sizes, shapes, and polymer compositions. In this study, we reported a maximum treatment time of 4 h and evaluated the cytotoxicity, proliferation, and ROS production only after 24 and 48 h. It is also crucial to evaluate the toxicity at extended times, chronic inflammation, and an immune response. On the other hand, it is also important to evaluate the motion while there is living tissue with blood flow and an immune response. Future work will focus on evaluating biofilm eradication using different bacterial strains and fungi like Candida, both individually and including epithelial cells, which will represent the real physiological condition.

## 3 Conclusion

This study is a first proof of concept for microplastic capture and biofilm eradication in simulated biological fluid using magnetic soft microrobots. Spherical soft microrobots were successfully fabricated by using a homogenizer-based droplet solidification method. This method provides the ability to tune the size of the soft microrobots by changing the injection speed and the rotation of the homogenizer. Soft microrobots incorporated with a contrast agent were successfully navigated using an external magnetic field and permanent magnets. Soft microrobots with a contrast agent were visible under X-ray fluoroscopy. Although the speed of the soft microrobots decreased when incorporated with a contrast agent, it was still sufficient to move the soft microrobots inside the rat uterine horns using a permanent magnet. The rate of microplastic capture in PBS was higher than that in SVF. However, it must be noted that bacterial capture and killing efficiency could be further enhanced by optimizing the size and injection dose of the soft microrobots as well as functionalization with antibiotics.

## 4 Methods

### 4.1 Fabrication of Magnetite (Fe_3_O_4_) Nanoparticles

Magnetite (Fe_3_O_4_) nanoparticles were used to control the external motion of the magnetic soft microrobots. It was prepared using a one-step hydrothermal process according to the following protocol.^30,31^ Briefly, 2.5 mmol of iron­(III) chloride hexahydrate (FeCl_3_·6H_2_O) was dissolved in 20 mL of ethylene glycol, followed by the addition of 1.8 mmol of sodium acetate and 0.5 g of polyethylene glycol (PEG 1000) as a surfactant. The mixture was vigorously stirred for 30 min to 1 h until a transparent solution was achieved. The final solution was then transferred into a 50 mL Teflon-lined stainless-steel autoclave, and the reaction proceeded at 200 °C for 12 h in the oven. After natural cooling to room temperature, the product was collected using a magnet and washed five times with ethanol, followed by drying at 60 °C.

### 4.2 Fabrication and Surface Functionalization of Magnetic Soft Microrobots

Magnetic soft microrobots consisting of magnetite, PDMS elastomer, curing agent (from Sylgard 184 (Sewang Hitech, Korea)), chitosan as an external motion control unit to maintain a soft scaffold in microrobot form, and an antibacterial agent were prepared using the droplet solidification process with an oil-in-water emulsion as shown in Figure 2a.^32^ 1% (w/v) chitosan with a molecular weight of 100,000–300,000 was dissolved in 2% (v/v) acetic acid (ACS grade). The solution was stirred for 24 h at room temperature until it was completely dissolved. A 1% chitosan solution (0.008 g), Fe_3_O_4_ nanoparticles (0.03 g), and 30% (w/w) tantalum metal were mixed with 0.5 g of PDMS and 0.05 g of cross-linker. Then, the mixture was loaded into a 1 mL syringe equipped with a 21G needle and injected into a 2% PVA solution at 80 °C via droplet-wise manual injection. Then, the soft microrobots were kept in the same 2% PVA solution at 60 °C overnight to enhance the cross-linking. Afterward, soft microrobots were collected and washed with water and ethyl alcohol twice. Then, the magnetic soft microrobots were coated with polydopamine via a self-polymerization process as described previously with minor modifications.^26,32,33^ Briefly, 10 mM Tris-HCl was prepared, 2 mg/mL of dopamine HCl was dissolved, and the pH was adjusted to 8.4 using 2 M NaOH. Afterward, 0.1 g of soft microrobots were added to 20 mL of PDA solution and shaken at 600 rpm for 1 day at room temperature. Polydopamine-coated soft microrobots were collected using a permanent magnet, washed twice with water, and dried in an oven at 60 °C. The adhesion behavior of PDA mimics the mussel protein.^34^

## 5 Characterization

Surface images of the soft microrobots were acquired by using a high-resolution SEM FEI Verios 460L in secondary electron (SE) mode at an accelerating voltage of 1 kV and a probe current of 13 pA. The images were analyzed by using ImageJ software.

FTIR measurement was conducted by utilizing vacuum FTIR Vertex70v-Attenuated Total Reflection (ATR) (Bruker, USA) with a diamond crystal. The resolution was set to 3 cm^–1^ while the sample scan time was 32 cm^–1^. Soft microrobots were dispersed on an ATR diamond for measurement of the sample peak. The protein corona formation was investigated by incubating the magnetic soft microrobots in FBS for 1 h, followed by collection and obtaining FTIR spectra.

### 5.1 Setup of Magnetic Actuation System

VSM measurements were acquired to investigate the magnetic properties of soft microrobots and contrast agent-loaded soft microrobots. Afterward, custom-made magnetic field generators, consisting of four orthogonal coil pairs in a poly­(lactic acid) (PLA) support, were used to generate a transverse rotating magnetic field. It was connected to the inverted microscope (Nikon ECLIPSE TS2R) with a digital camera (Basler acA1920–155uc) to record the motion behavior of the microrobots in different media (PBS and SVF) as illustrated in the Supporting Information Figure S3. Ten μL of the microplastic and soft microrobot mixture was dropped on a glass slide, and the microplastic and soft microrobot interaction and navigation were recorded under a 5 mT magnetic field with the frequency of 10 mHz. The recorded videos were analyzed with Fiji software. The motion behavior of the soft microrobots has also been evaluated in the presence of contrast agents. Microplastic capturing was examined in SVF. The magnetic locomotion of the soft microrobots was achieved using a permanent NdFeB magnet in a phantom model of the female rat reproductive system. In this experiment, magnetic soft microrobots containing contrast agents were injected into the uterine horns, and magnets were placed near the organ and moved from left to right.

### 5.2 Quantification of Microplastic Removal

A yellow-green, fluorescence-tagged carboxylate-modified polystyrene (Sigma-Aldrich) with a size of 2–3 μm was selected to demonstrate microplastic capture in two different media, including 1× PBS and SVF, to demonstrate different ionic strength and pH conditions in pH 7.4 and 4.6, which simulates a female reproductive system pH condition. Simulated vaginal fluid was prepared following the Owen and Katz protocol.^35,36^ Briefly, it was prepared using 3.51 g/L of sodium chloride, 1.4 g/L of potassium hydroxide, 0.22 g/L of calcium hydroxide, 0.018 g/L of bovine serum albumin, 2.0 g/L of lactic acid, 1 g/L of acetic acid, 0.16 g/L of glycerol, 0.4 g/L of urea, and 5.0 g/L of glucose. Twenty-five μL of the stock microplastic solution was diluted with 20 mL of medium to form a stock solution, and 2 mL of this solution containing 8.24 × 10^6^ particles was treated with 0.02 g of soft microrobots. Each experiment was repeated 4 times for 1× PBS and SVF, with a time-dependent capture experiment conducted by treating microplastic-containing media for 30 min, 1, 2, and 4 h. The permanent magnets were placed at the same distance as the samples, which were shaken using orbital shakers at a rate of 200 rpm during the treatment. After that, soft microrobots were collected from the microplastic solution using magnets. A Jasco FP8300 spectrofluorometer was used to measure the fluorescence emission spectra of the remaining microplastics in the medium compared with the positive control. All measurements were recorded at the excitation wavelength of 470 nm. The collected soft microrobots were then visually characterized using an SEM image to verify captured microplastics on the soft microrobots.

### 5.3 Evaluation of Dead Bacteria Using Live/Dead Bacterial Staining

To evaluate the killing effect on the surface of the magnetic soft microrobots, we obtained fluorescence images after LIVE/DEAD staining (LIVE/DEAD BacLight Bacterial Viability Kits, Invitrogen) using SYTO 9 and propidium iodide (PI). Briefly, 1 × 10^8^ CFU/mL of *E. coli* cells were cultured in LB broth, and 200 μL of the suspension was transferred to a 96-well plate. Afterward, microrobots at a concentration of 10 mg/mL were added to each well. Microrobots without bacteria and microrobots without a polydopamine coating were used as control groups to assess any interference between the staining and the microrobots and the antibacterial effect of the polydopamine layer. Each well plate was treated for different time intervals, including 30 min, 1 h, 2 h, and 4 h, while the two control groups were treated for 4 h. Bacterial suspensions in each well were removed, and remaining microrobots were washed gently with deionized (DI) water prior to the staining protocol. Excess dye was removed by washing with DI water again. Afterward, bright-field and fluorescence images were acquired using a Zeiss LSM 980 confocal microscope with a Plan-Apochromat 10×/0.45 NA objective. Confocal micrographs are shown in separate channels for live (green) and dead (red) cells.

### 5.4 Biofilm Eradication

*E. coli* (Czech Collection of Microorganisms (CCM), Brno, Czech Republic) was used to demonstrate biofilm eradication. The bacteria were grown in Luria−Bertani (LB) broth to an optical density of 0.5 at 590 nm (OD590). Then, the bacterial suspension (200 μL) was seeded into a clear-bottomed 96-well plate and incubated for 24 h at 37 °C. LB broth was replenished 24 h later, and the incubation continued at 37 °C. Following the incubation period, bacterial biofilms were washed twice with PBS. The next day, the LB broth was replenished, and soft microrobots at a concentration of 10 mg/mL were added to each well. Two permanent magnets were placed on the orbital shaker, and a 96-well plate was mounted on a permanent platform 1 cm above the shaker. The biofilm eradication experiment was conducted for different treatment durations, with treatment durations of 0, 30 min, 1, 2, 3, and 4 h; the untreated sample at 0 h served as the positive control. After treatment, the microrobots were removed, and the plates were washed once with PBS. Each well was stained using 200 μL of 0.1% (v/v) Crystal Violet staining solution and incubated at 37 °C for 5 min. Next, the stain was removed and the microrobots were rewashed with 1× PBS before the wells were incubated with 30% (v/v) acetic acid for 30 min to dissolve the bound Crystal Violet. Then, the acetic acid solution was transferred to clean wells, and the optical density was measured at a wavelength of 590 nm. A heatmap of the remaining biofilm was obtained using an absorbance plate reader (800 TS microplate reader) and a SPECTROstar Nano. The experiment utilized SPECTROstar Nano MARS and Prism Version 9.5.1 software for measurement, data analysis, and statistics.

### 5.5 Real-Time Visualization by Medical Image Guidance

The contrast agent, consisting of microsized tantalum, was chosen and incorporated with soft microrobots for real-time visualization. X-ray fluoroscopy was performed using an Allura Xper FD 10 R8 Niobe (Philips) for soft microrobots in the syringe and a 9800 Plus Cardiac C-arm system (GE Healthcare) for measuring the phantom female rat reproductive system. First, a 1 mL syringe was filled with soft microrobots incorporated with a contrast agent, 0.9% saline solution as a negative control, and 30% (w/v) tantalum nanoparticles in saline as a positive control. Afterward, different tantalum-containing soft magnetic microrobots were evaluated inside the 1 mL syringe using the same protocol. All syringe images were obtained by applying a voltage of 51 kV and a 50.0 mA current. For the female rat reproductive system, a 15-mA current and 48 kV voltage were applied to demonstrate the real-time visualization. Color intensity was analyzed by using ImageJ software.

### 5.6 Cell Culture and MTT Assay

Human fibrosarcoma epithelial-like cell line (HT1080) and human embryonic kidney (HEK-293) cell line were purchased from the American Type Culture Collection (ATCC, USA) and were used for cell cytotoxicity evaluation. HT1080 cells were grown in minimum essential medium (MEM) supplemented with nonessential amino acids (0.1 mM), 10% FBS, sodium pyruvate (1 mM), l-glutamine (2 mM), and gentamicin (10 μg/mL), while HEK-293 cells were grown in DMEM F12 medium supplemented with 100 U/mL penicillin, 100 μg/mL streptomycin, and 10% FBS. Cells were incubated and grown until ∼80–90% confluence in a humidified incubator at 37 °C in 5% CO_2_ atmosphere for 24 h. All cell lines were tested for mycoplasma infection (MycoAlert Mycoplasma Detection Kit, Lonza, Switzerland) according to the manufacturer’s protocol. Untreated cells were used as control groups for each biological assay. Cell viability was evaluated using 3-(4,5-dimethylthiazol-2-yl)-2,5-diphenyltetrazolium bromide (MTT) reduction assays according to the manufacturer’s protocol. Briefly, 4 × 10^3^ HT1080 cells and 10 × 10^3^ HEK-293 cells per well were inoculated in a clear-bottom 96-well plate for 24 h. The following day, the cells were washed with PBS and treated with 4 different concentrations of microrobots, 0.002, 0.004, 0.006, and 0.008 g per well for 24 and 48 h with 200 μL medium. For the assay, 5 mg/mL of the MTT reagent was dissolved in PBS. The reagent was then mixed with the cell culture medium to reach the final concentration of 0.5 mg/mL. The original medium was removed from the plates, and 100 μL of the medium with the MTT reagent was added to each well. Afterward, the absorbance values at 490 nm were recorded using a plate reader (SPECTROstar Nano).

### 5.7 ATP-Dependent Cell Viability Assay

10,000 cells/well were seeded in a 96-well plate. After reaching 80% confluence, cells were treated with microrobots and control NPs and incubated according to selected time points. After incubation, Cell Titer Glo Luminescent Cell Viability Assay (#G7571, Promega, USA) was used for ATP-Dependent Cell Viability Assay. The reagent was prepared according to official protocol and was applied to the cells in a volume of 100 μL/well. Plates were shaken at an orbital shaker for 2 min to ensure proper mixing of the Cell Titer Glo reagent with the medium. Afterward, cells were incubated at RT for 10 min to stabilize the luminescence for the measurement. The whole volume from each well was then transferred into a white 96-well plate, and luminescence was measured using a SYNERGY H1 microplate reader.

### 5.8 Cell Proliferation

10,000 cells/well were seeded in a 96-well plate. After reaching 80% confluence, cells were treated with microrobots and incubated at the selected time points. Untreated HEK-293 cells were used as controls. Cell proliferation ELISA BrdU colorimetric assay kit (#11647229001) was used and followed the protocol provided by Roche (Sigma-Aldrich, USA). Briefly, cells were cultured in 100 μL/well of cell culture medium. Ten μL of BrdU labeling solution (final concentration 10 μM BrdU) was added to each well. Cells were incubated for 2 h at 37 °C. After the incubation, the medium with BrdU labeling solution was gently removed, and the microplates were placed at 60 °C to dry for 60 min. After drying, 200 μL of FixDenat solution/well was added. Plates were incubated for 30 min at RT. After the incubation, the FixDenat solution was gently removed, and 100 μL of Anti-BrdU-POD working solution was added to each well. Plates were incubated for 90 min at RT. The antibody conjugates were removed from the wells by flicking them off, and each well was rinsed with 200 μL of PBS three times. After rinsing the wells, 100 μL of Substrate Solution was added to each well and cells were incubated at RT until the color development was sufficient for photometric detection (approximately 10 min). Afterward, the absorbance of samples and the blank was measured in a Tecan Infinite 200 PRO spectrophotometer at 370 nm (reference wavelength 492 nm).

### 5.9 ROS Induction Assay

10,000 cells/well were seeded in a black, clear-bottom 96-well plate. After reaching 80% confluence, cells were treated with microrobots and incubated for 24 h. After incubation, cells were washed with 200 μL of warm PBS. Subsequently, CellROX Green (#C10444, Thermo Fisher Scientific, USA) was added to the cells. The CellROX Green working solution was prepared by diluting the CellROX Green stock solution at a ratio of 1:500 (v:v) CellROX Green: PBS. Cells were incubated for 30 min at 37 °C, and H_2_O_2_ was used as a positive control. After incubation, each well was rinsed with 200 μL PBS. Fluorescence was measured at ex./em. 485/520 nm.

### 5.10 Agarose Gel Electrophoresis for Protein Corona Formation

Microrobots at various concentrations (40, 30, 20, 10 μg/mL) were incubated with FBS for selected periods of time at the rotation speed of 450 rpm at 37 °C. After incubation, samples were centrifuged at 15,000 rcf for 10 min. After the first centrifugation, microrobots formed a pellet. The supernatant was gently removed, and samples were resuspended in fresh PBS. This step was repeated 5 times. After the last centrifugation, the samples were resuspended in 50 μL PBS. To visualize the protein corona, a 1% agarose gel (molecular biology grade, #11380.03, SERVA Electrophoresis GmbH, Germany) was prepared. To load samples onto the gel, 20 μL of the sample was mixed with 3 μL of glycerol. FBS and an unstained protein ladder (10–250 kDa, #26614, Thermo Fisher Scientific, USA) were used for comparison. The electrophoresis was set at 85 V for 35 min. To visualize protein coronas, a cascade of Coomassie blue staining was used. The gel was subsequently visualized using Azure c600.

### 5.11 Ethics Statement

Part of the research connected with testing nanorobots in the rat uterus was conducted at the Veterinary Research Institute in Brno, which is fully certified for research on animal models (accreditation nos. MZE-778/2025, MZE-18771/2025-13143). The actuation and imaging of the magnetic soft microrobots in the rat uterine horn experiment were performed exclusively on donated rat uterine horn tissues obtained after euthanasia for unrelated purposes, and no animals were sacrificed specifically for this work.

## Supplementary Material















## Data Availability

The data that support the findings of this study are available from the corresponding author upon reasonable request.

## Notes

An illustration of the fabrication method; magnetic hysteresis loop of magnetic soft microrobots; an additional SEM image of magnetic soft microrobot with bacteria on it; heatmap profiling of biofilm on a 96-well plate; an illustration of the X-ray fluoroscopy imaging used for a clinically relevant imaging method; and cell viability of the bare magnetite nanoparticles before incorporation into the soft microrobots (PDF)
Actuation of magnetic soft microrobots in rat uterine horn (Supporting Movie 1) (MP4)
Magnetic soft microrobots interaction with microplastics in PBS (Supporting Movie 2) (MP4)
Magnetic soft microrobots interaction with microplastics in SVF (Supporting Movie 3) (MP4)
Trajectory of the magnetic soft microrobots captured microfibers (Supporting Movie 4) (MP4)
Retrieval of magnetic soft microrobots 1 (Supporting Movie 5) (MP4)
Retrieval of the magnetic soft microrobot 2 (Supporting Movie 6) (MP4)
