# Supplementary material for: Image-Guided Magnetic Microrobots for Microplastic Capture and Biofilm Removal in the Female Reproductive System
Source: ACS Nano. 2026 Sep 3;20(37):25264–74. doi: 10.1021/acsnano.6c02432 (PMC13602912; doi:10.1021/acsnano.6c02432)
Supplement: Supplementary file 1 [file nn6c02432_si_001.pdf]

Image-Guided Magnetic Microrobots for Microplastic Capture and Biofilm Removal in the Female Reproductive System

Fatma M. Yurtsever,[a] *Sagar Arya,*[a] *Ester Maráková,*[b] *Roshan Velluvakandy,*[a] *Eva Odehnalová,*[c] *Jakub Hejč,*[c] *Zbyněk Heger,*[b,d] *Martin Pumera*[a,e,f,g]*

[a] Future Energy and Innovation Laboratory, Central European Institute of Technology, Brno University of Technology, Purkyňova 123, Brno, 61200, Czech Republic

[b] Department of Chemistry and Biochemistry, Mendel University in Brno, Zemědělská 1665/1, Brno, 613 00, Czech Republic

[c] International Clinical Research Center, St. Anne's University Hospital Brno, Pekařská 664/53 | 602 00 Brno | Czech Republic

[d] Center of Advanced Innovation Technologies, Faculty of Materials Science and Technology, VSB - Technical University of Ostrava, Ostrava, CZ-708 00, Czech Republic

[e] Department of Medical Research, China Medical University Hospital, China Medical University, No. 91 Hsueh-Shih Road, Taichung, 40402, Taiwan

[f] Office of Research and Development, Asia University, Taichung 40402, Taiwan

[g] Faculty of Electrical Engineering and Computer Science, VSB - Technical University of Ostrava, 17. listopadu 2172/15, 70800 Ostrava, Czech Republic

*M.Pumera, email: martin.pumera@ceitec.vutbr.cz

Content

Figure S1: Illustration of magnetic soft microrobot fabrication method.

Figure S2: Magnetic hysteresis loop of magnetic soft microrobots.

Figure S3: Illustration of custom-built magnetic field generator setup and trajectory of the magnetic soft microrobots.

Figure S4: SEM images of the soft microrobot with bacteria on it.

Figure S5: Heatmap profile of the remaining biofilm on the bottom

Figure S6: Demonstration of medical imaging of the magnetic soft microrobots using an X-ray fluoroscopy.

Figure S7: Cell viability of HEK-293 cell line after incubation with magnetite nanoparticles

Supporting Movie 1: Actuation of magnetic soft microrobots in rat uterine horn

Supporting Movie 2: Magnetic soft microrobots interaction with microplastics in PBS

Supporting Movie 3: Magnetic soft microrobots interaction with microplastics in SVF

Supporting Movie 4: Trajectory of the Magnetic Soft Microrobots Captured Microfibers

Supporting Movie 5: Retrieval of magnetic soft microrobots 1

Supporting Movie 6: Retrieval of the Magnetic Soft Microrobot 2

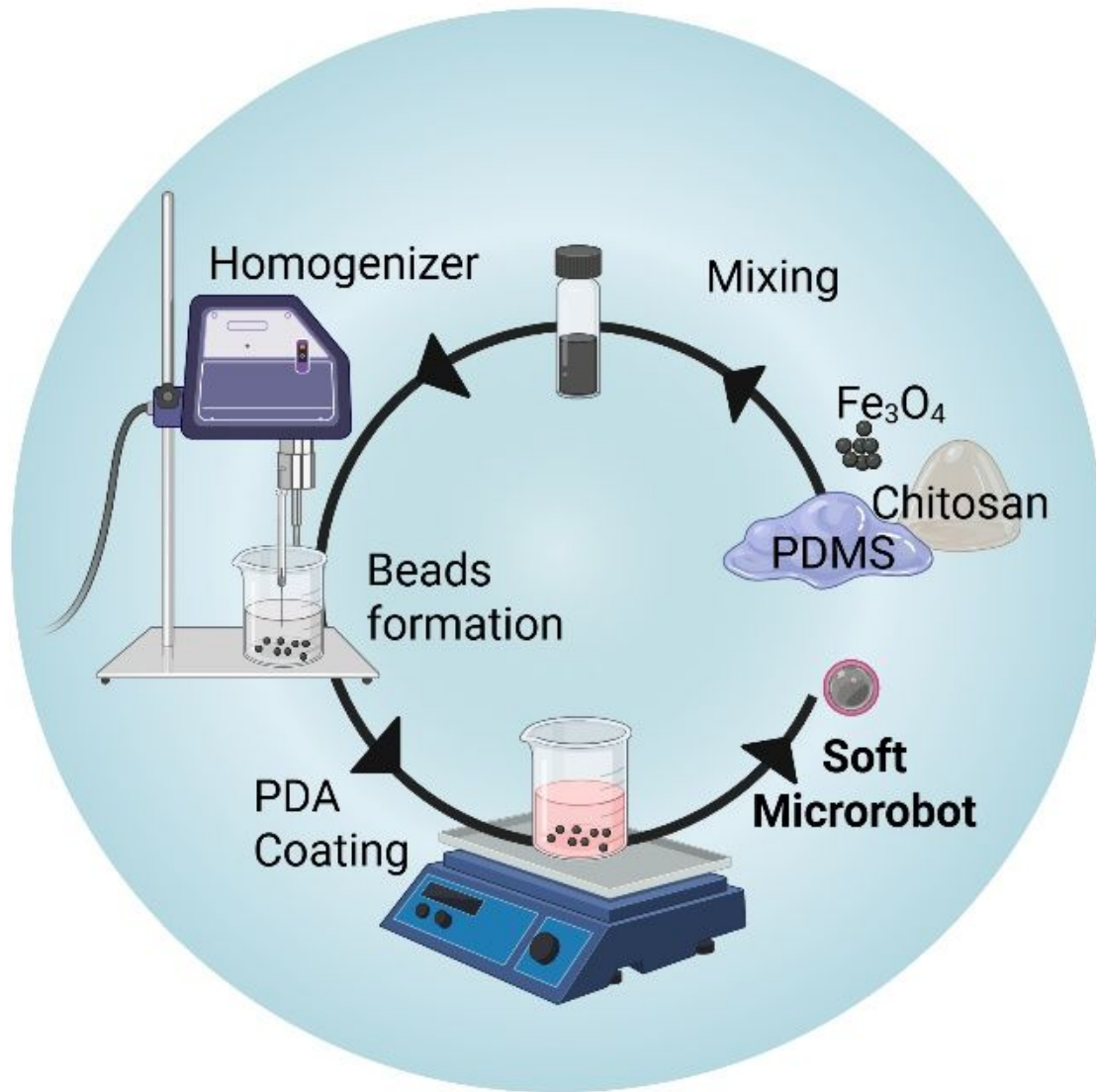

Figure S1. Illustration of magnetic soft microrobot fabrication by using droplet solidification process with oil-in-water emulsion, followed by polydopamine (PDA) coating. Ilustration was prepared with BioRender.com.

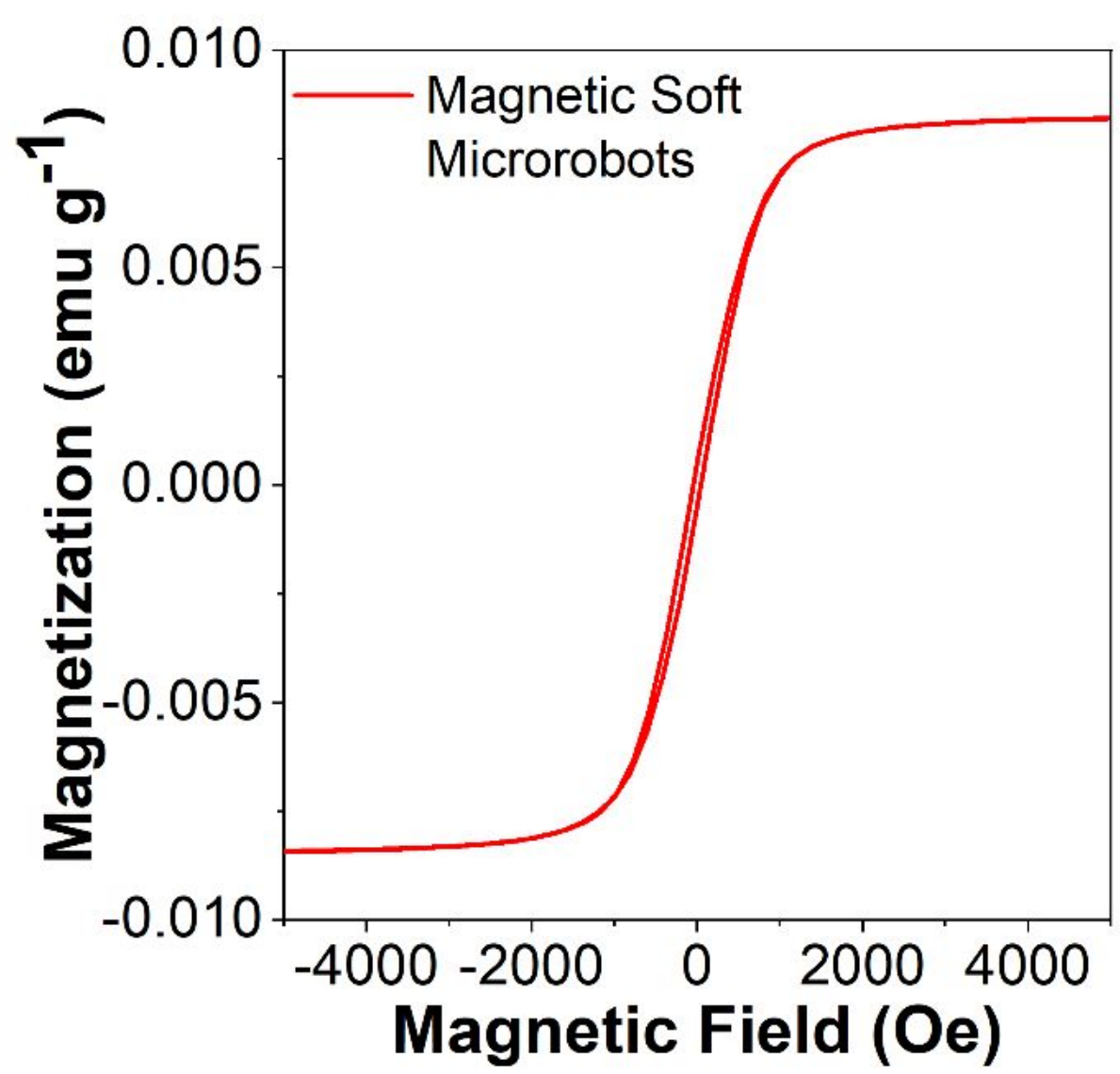

Figure S2. Magnetic hysteresis loop of magnetic soft microrobots indicating the superparamagnetic behavior.

Magnetic Actuation Setup

Magnetic actuation tests and analysis were conducted using the previously demonstrated method.[1] Briefly, the custom-made magnetic field generator connected to the three orthogonal coil box placed in an inverted optical microscope (Nikon Ts2R) connected to a digital camera (Basler acA1920-155uc). Pylon Viewer software at 25 fps was used to record the videos, and the motions were analyzed using Fiji software. The transversal rotating magnetic field **B** (B_x, B_y, B_z) are stated by the following equations:

$$B_x = B_0 \cos(\omega t) \sin(\alpha)$$

$$B_y = B_0 \cos(\omega t) \cos(\alpha)$$

$$B_z = B_0 \sin(\omega t)$$

B_0 is the magnetic field amplitude, proportional to the coils' current ω (ω= 2π*f*), *f* is the frequency [Hz], *t* is the time in seconds, and α is the navigation angle (0–360°). The direction of the microrobots was tuned by manipulating α on the *xy* plane, allowing their precise navigation.

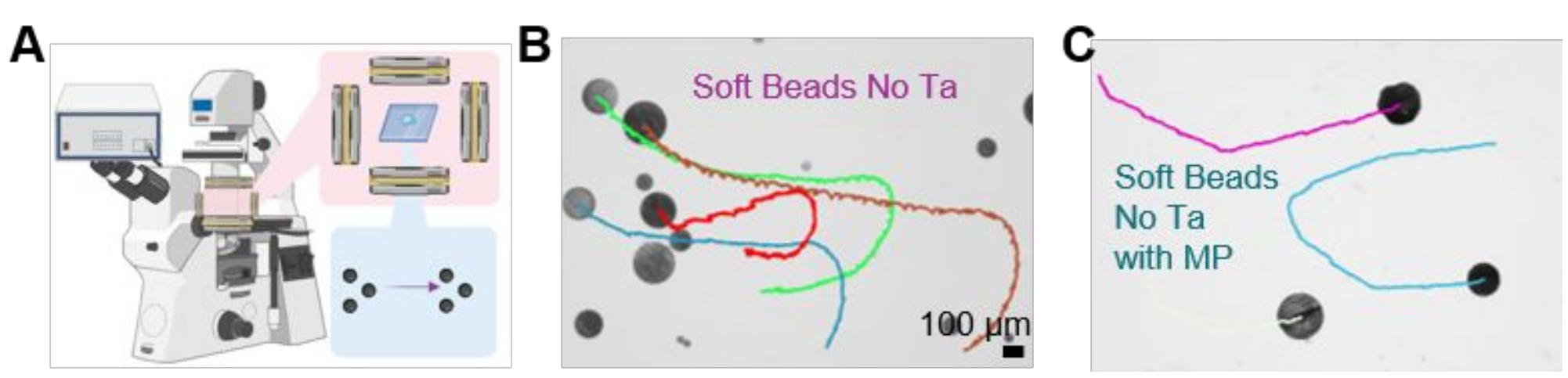

Figure S3. A) Illustration of custom-built magnetic field generator connected with inverted optical microscope. Illustration was prepared with BioRender.com. Motion tracking of the magnetic soft microrobots, B) without contrast agent, C) without contrast agent and with microplastic in the medium.

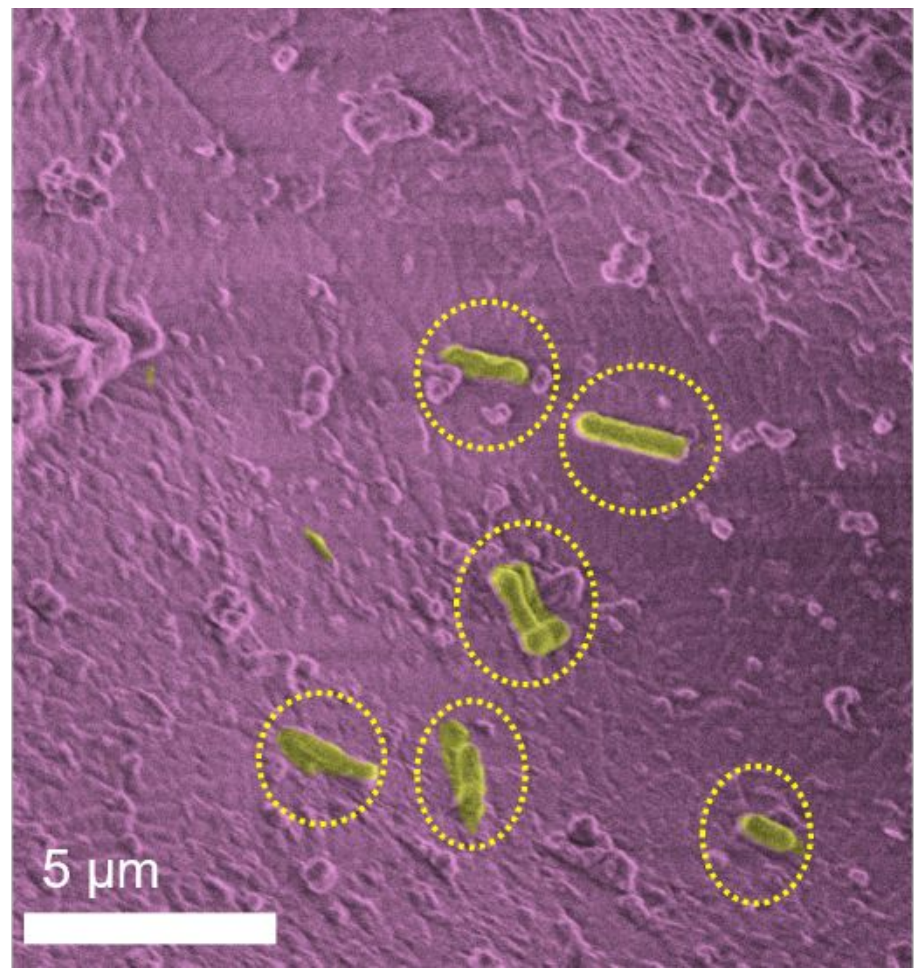

Figure S4. SEM images of the soft microrobot surface after capturing *E. Coli.* (Bacteria were represented with yellow circles and colors, and magnetic soft microrobots were represented with purple color. The image is artificially colored. The scale bar is 5 µm.

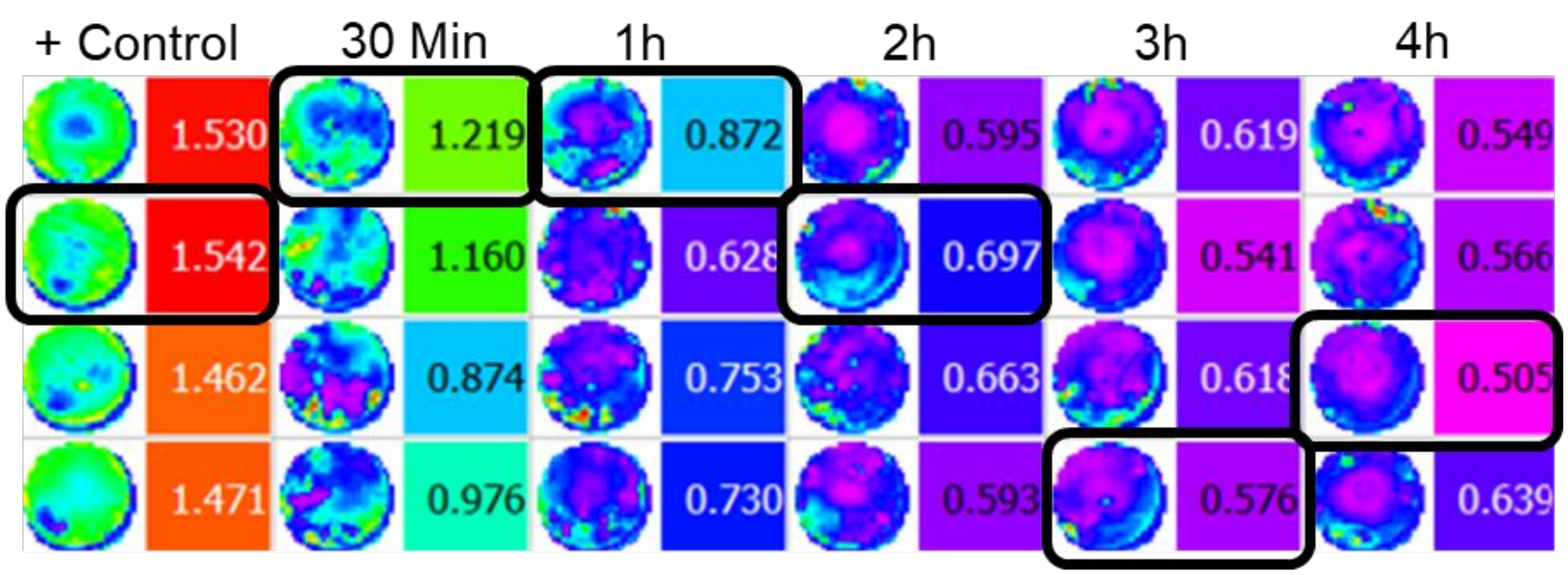

Figure S5. Biofilm eradication studies. Heatmap profile of the remaining biofilm on the bottom of the well after crystal violet staining. based on absorbance measurement at the wavelength of 590 nm. Black boxes indicate the representative images in Figure 4b.

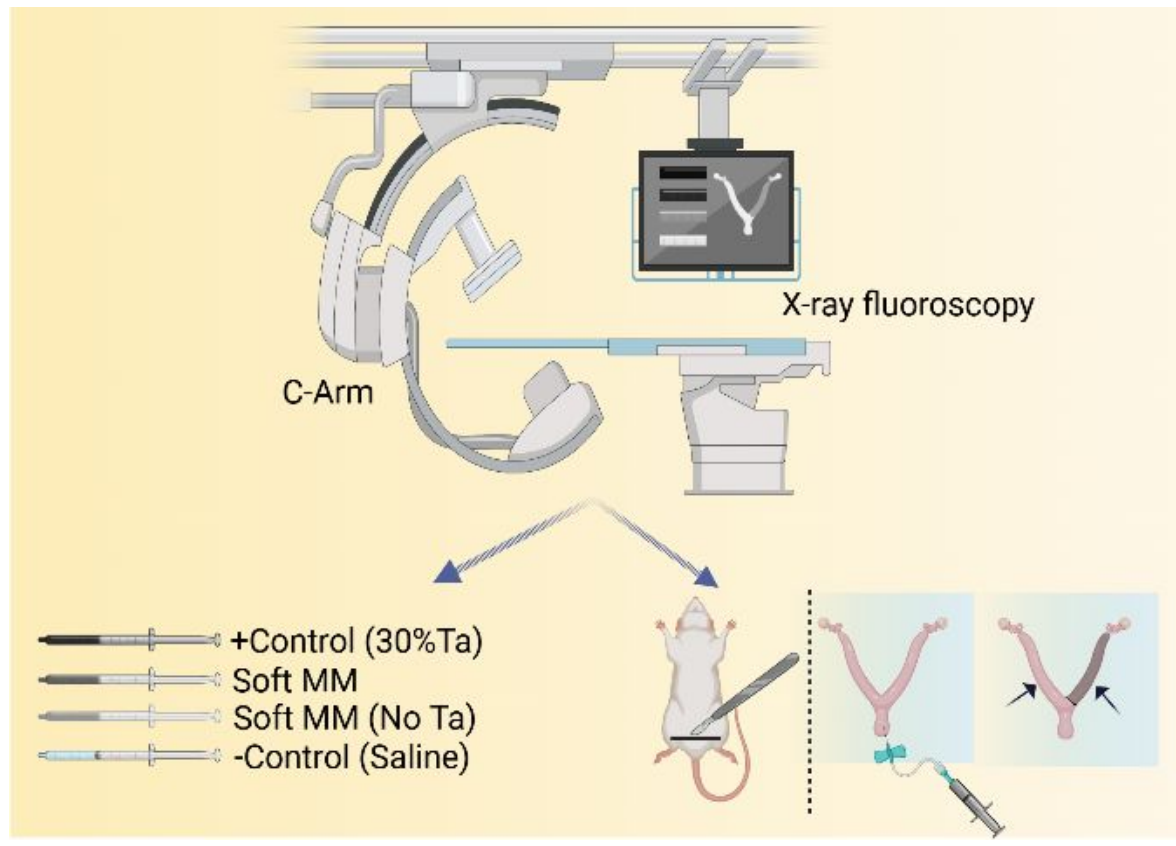

Figure S6. Demonstration of the real-time imaging of the magnetic soft microrobots using an X-ray fluoroscopy setup. Illustration was prepared with BioRender.com.

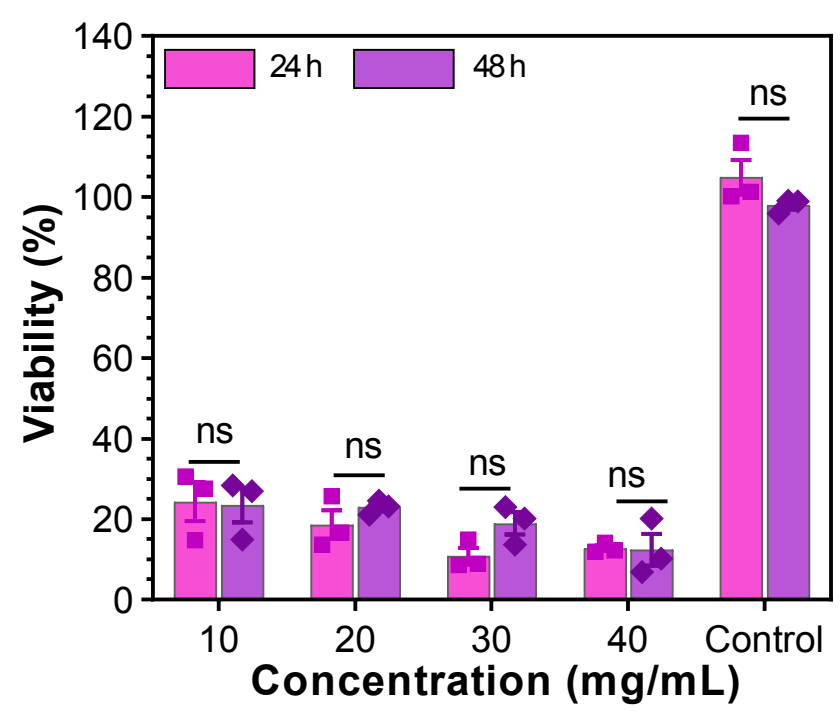

Figure S7. Viability of HEK-293 cell line after incubation with magnetite nanoparticles.

Reference

(1) Ussia, M.; Urso, M.; Oral, C. M.; Peng, X.; Pumera, M. Magnetic Microrobot Swarms with Polymeric Hands Catching Bacteria and Microplastics in Water. *ACS Nano* **2024**, *18* (20), 13171–13183.
